# Supplementary figures and images for: Schooling of light reflecting fish
Source: PLoS One. 2023 Jul 21;18(7):e0289026. doi: 10.1371/journal.pone.0289026 (PMC10361475; doi:10.1371/journal.pone.0289026)

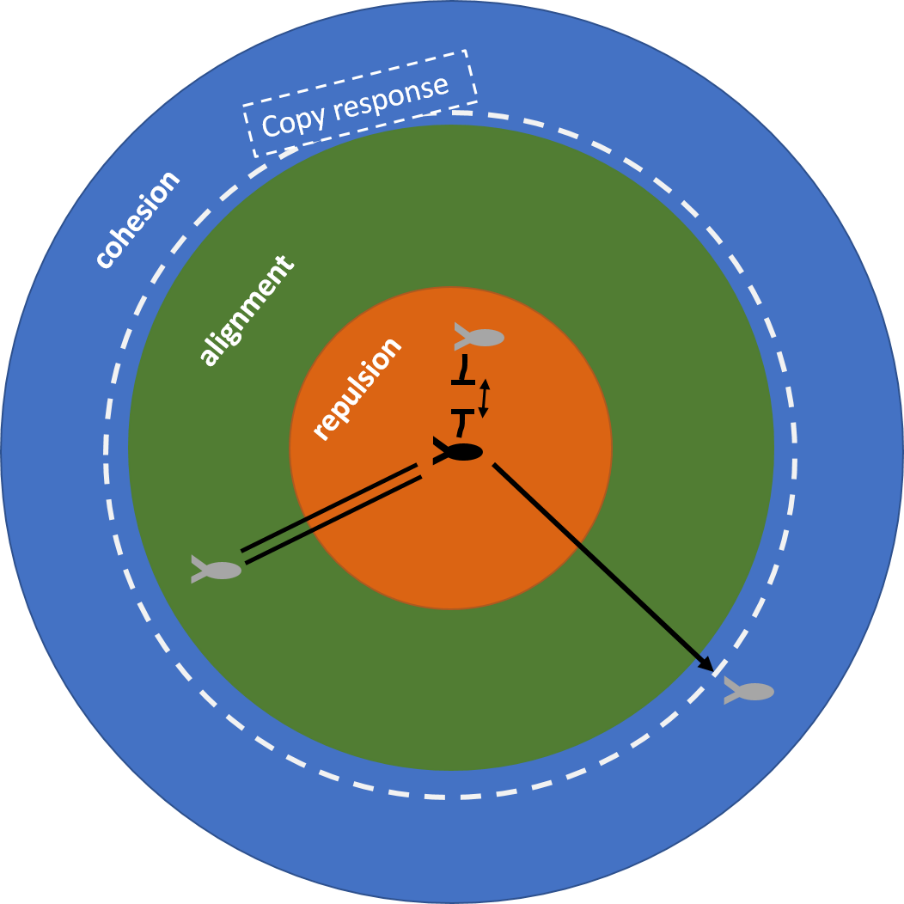

Supplement: S1 Fig — The agent (the black fish in the middle) modifies its motion based on its neighbors’ velocities and locations within the Cohesion zone (the agent is attracted towards the center of the mass of the neighbors’ position within this zone, which is typically the largest). The Alignment zone (the agent adjusts its direction towards the average direction of its neighbors within this zone) and the Repulsion zone (the agent tries to get away of the agents in this zone by swimming away of their center of mass weighted by their relative distance from the agents’ position. This zone, out of all zones, is typically the smallest and of the highest weight). Fish within the copy zone look for informed neighbors, whose swimming speed and direction they copy. Our default for this zone is the same as the alignment, assuming that from this distance the fish can clearly see changes in orientation. (TIF) [file pone.0289026.s002.tif]

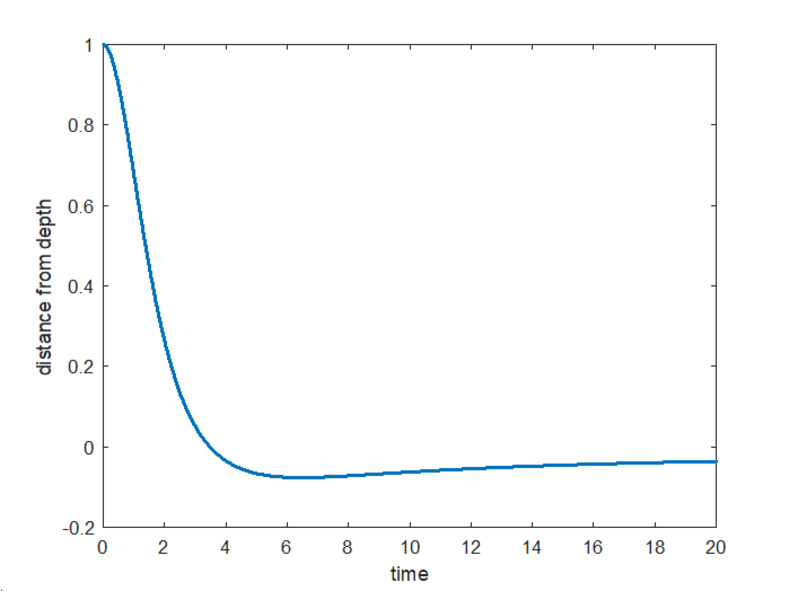

Supplement: S2 Fig — (TIF) [file pone.0289026.s003.tif]

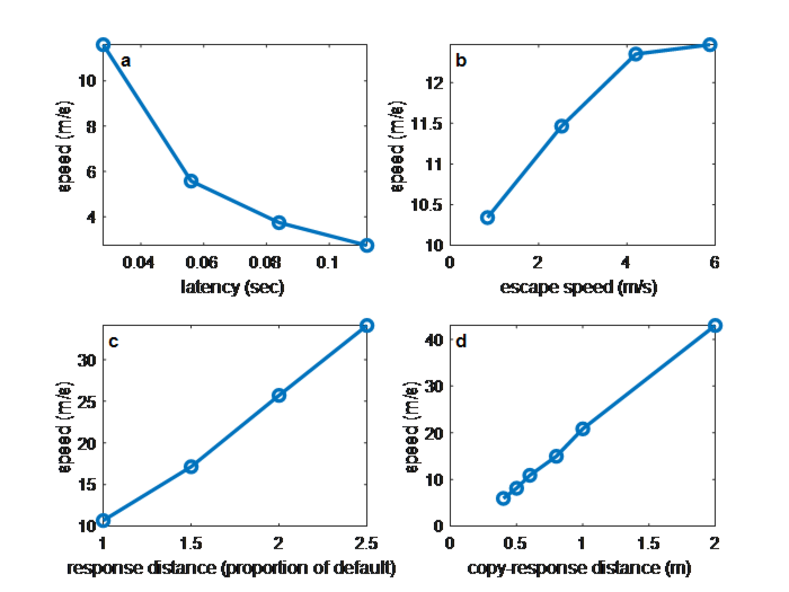

Supplement: S3 Fig — Wave speed as a function of parameters: a. latency, b. escape speed, c. response distances of all the motion rules (see default values in S1 Table in S1 File), d. the distance at which the fish applies the copy-response. (TIF) [file pone.0289026.s004.tif]

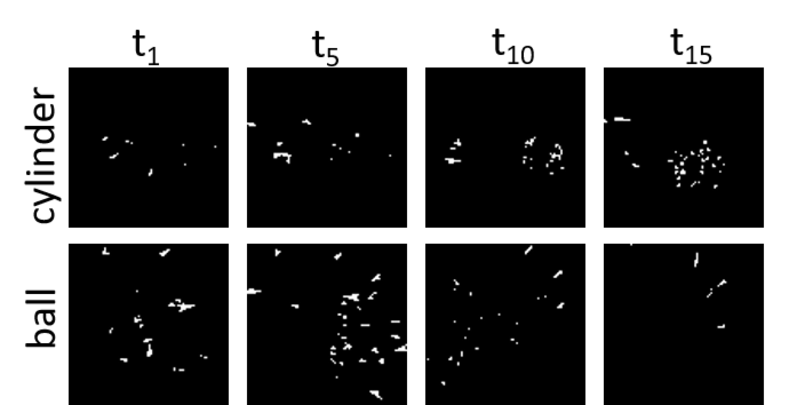

Supplement: S4 Fig — The attack came from the opposite direction to the school movement. The observer does not ‘know’ its position relative to the school (based on the flashes only) on the horizontal plane, and the center of the field of view of the observer is not fixed. Some of the flashes are due to random movement of the fish around their roll axis, while other are due to evasive or copy responses. (TIF) [file pone.0289026.s005.tif]

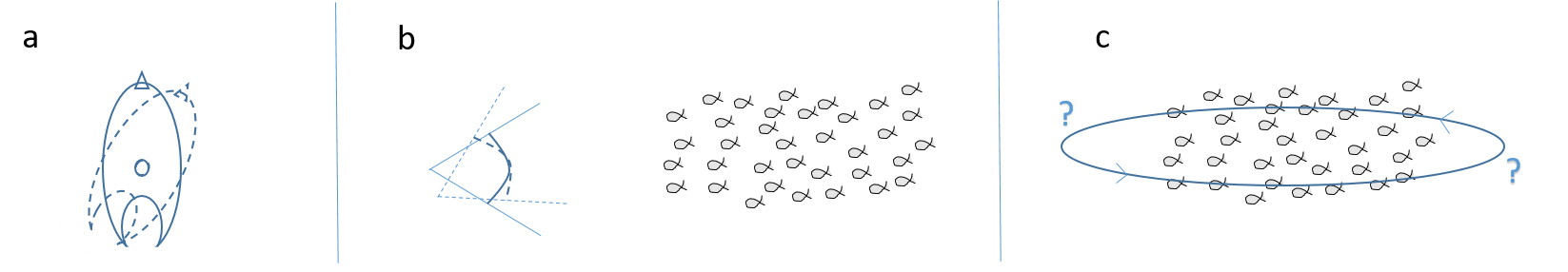

Supplement: S5 Fig — a. noise in the motion of the fish around the roll axis. b. noise in the look-at point of the observer. c. noise in the location of the observer on the horizontal plane around the school. (TIF) [file pone.0289026.s006.tif]

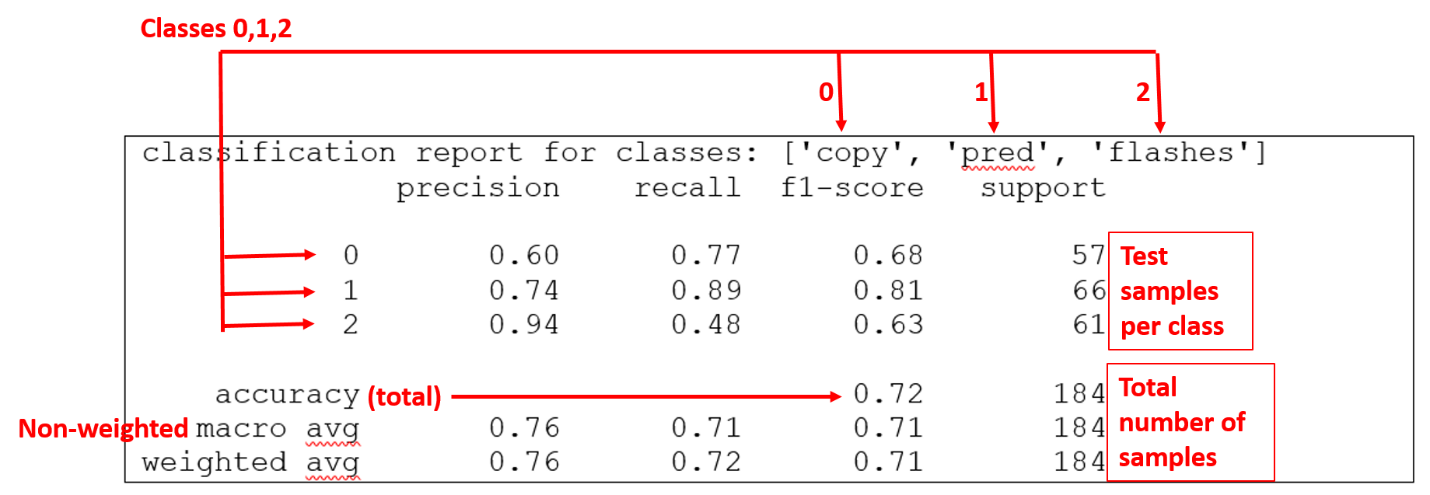

Supplement: S6 Fig — The names of the classes are presented in the top row by the order they appear in the table. For each class we measure the precision, recall, and f1-score. The total accuracy is being calculated for the entire model. Additionally, the support column presents the number of samples we used for testing each class. The last two rows present the weighted, and the non-weighted averages for precision, recall and f-score for the entire model. Since our dataset is balanced, the averaged f1-score is similar to the total accuracy, and there is not much difference between the weighted and the non-weighed averages. (TIF) [file pone.0289026.s007.tif]

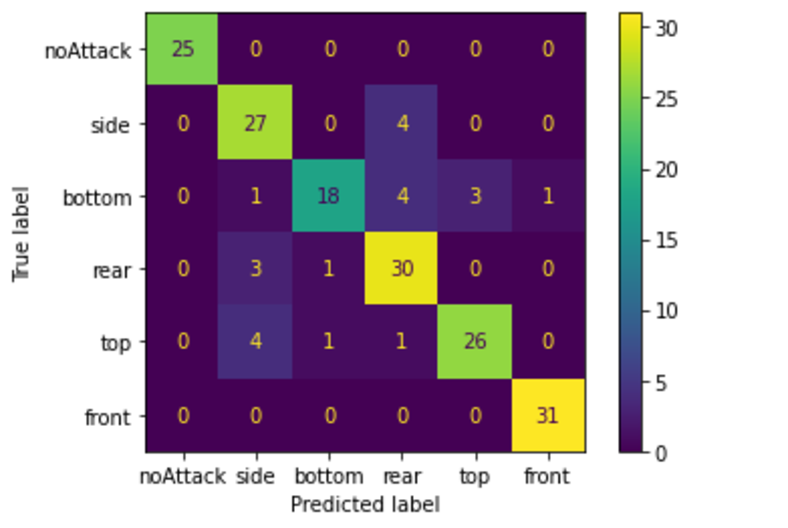

Supplement: S7 Fig — (TIF) [file pone.0289026.s008.tif]

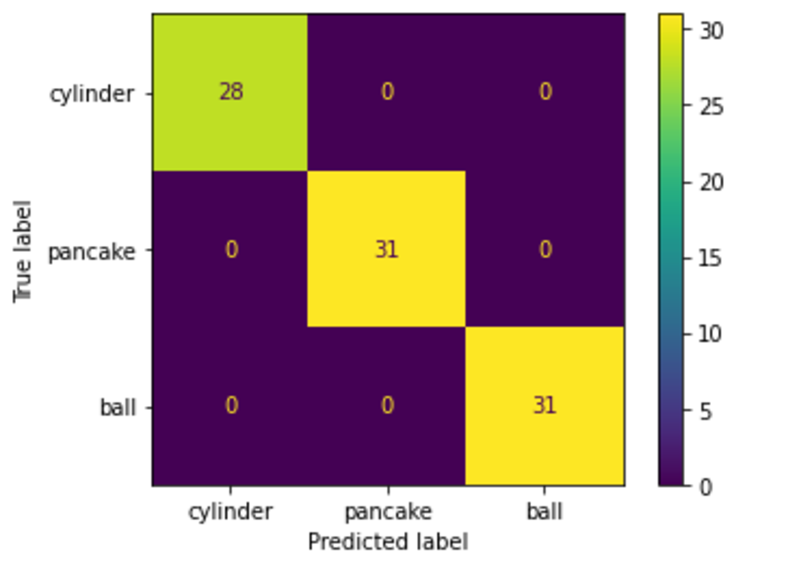

Supplement: S8 Fig — (TIF) [file pone.0289026.s009.tif]

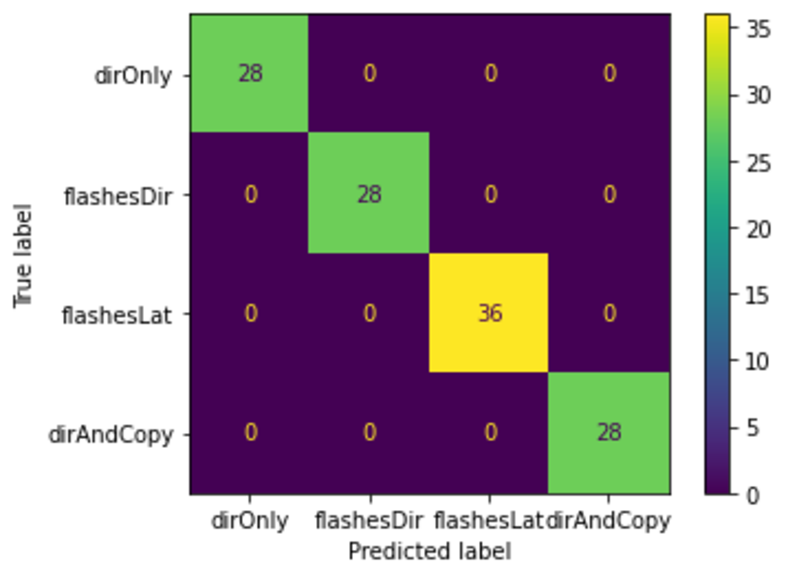

Supplement: S9 Fig — (TIF) [file pone.0289026.s010.tif]

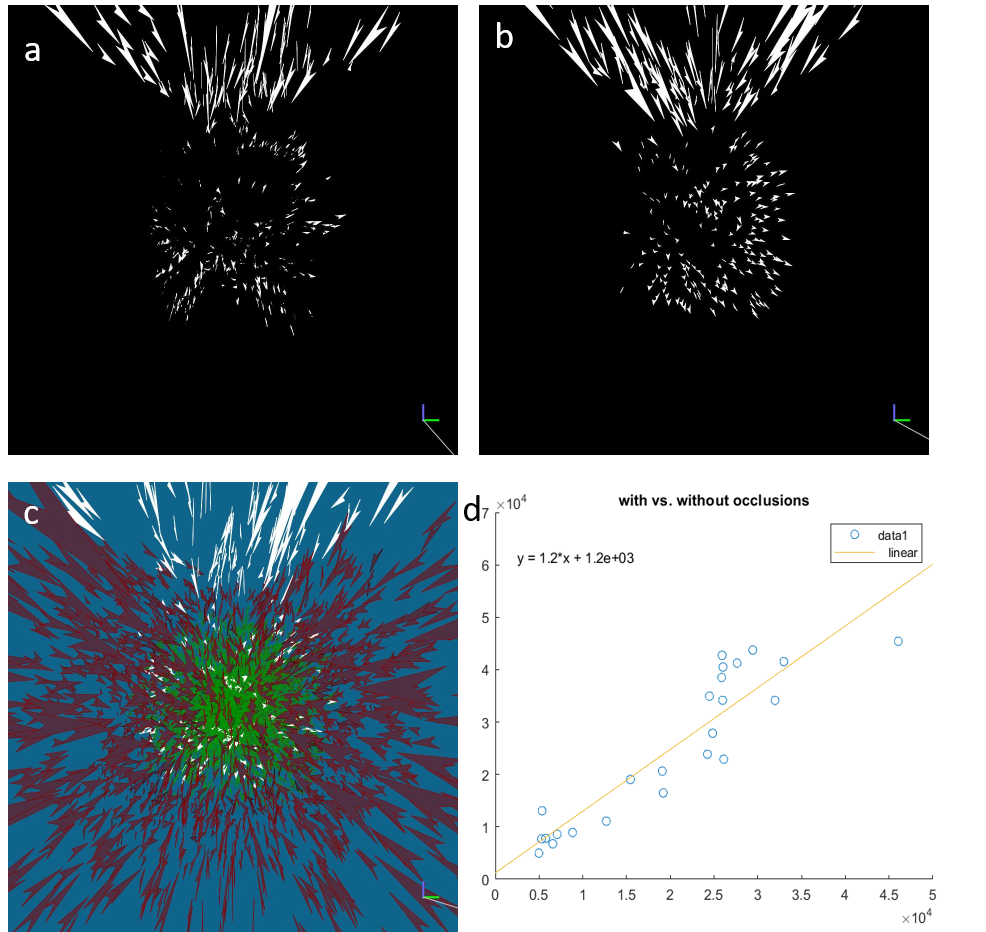

Supplement: S10 Fig — a. The flashes the observer sees on time t+20 with occlusions. b. The flashes the observer sees without occlusions. c. The “real” image the observer sees. Note that the red and the green are visual aids for us indicating whether the fish turning towards the observer or away of it. They are not “seen” by the observer. Also, the image here is not limited by the distance the observer “sees” when it calculates its Boids rules. d. Correlation between the changes in total area of the flashes with and without occlusions in the different steps. Each plot (and dataset) was calculated on a different run but is replicable. It is also important to note that in this simulation the fish are two-dimensional and in real three dimensional schools the effect of occlusion may be stronger and depend in the school density. (TIF) [file pone.0289026.s011.tif]

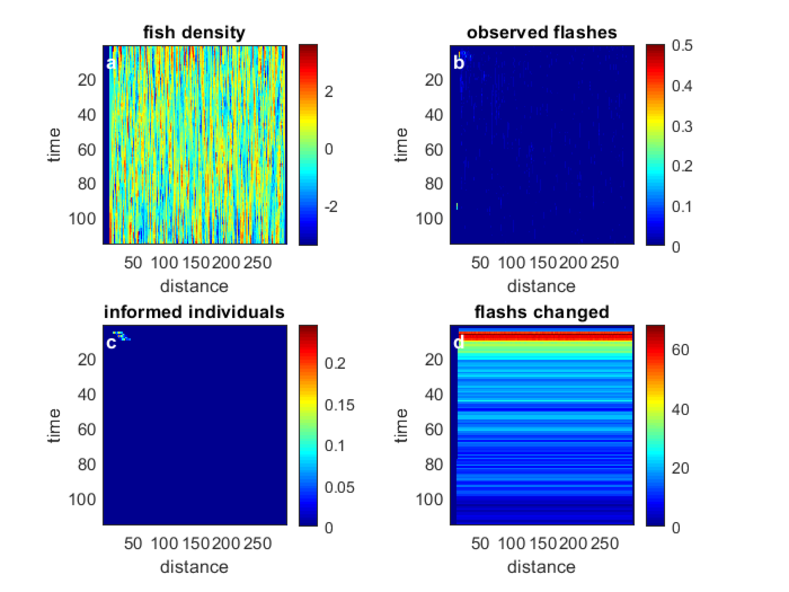

Supplement: S11 Fig — The only pattern that could be clearly detected is the local predation event on the left side of the school at the beginning of the scenario. On plot d we can see that this event generated a weak flash signal towards the entire school. (TIF) [file pone.0289026.s012.tif]

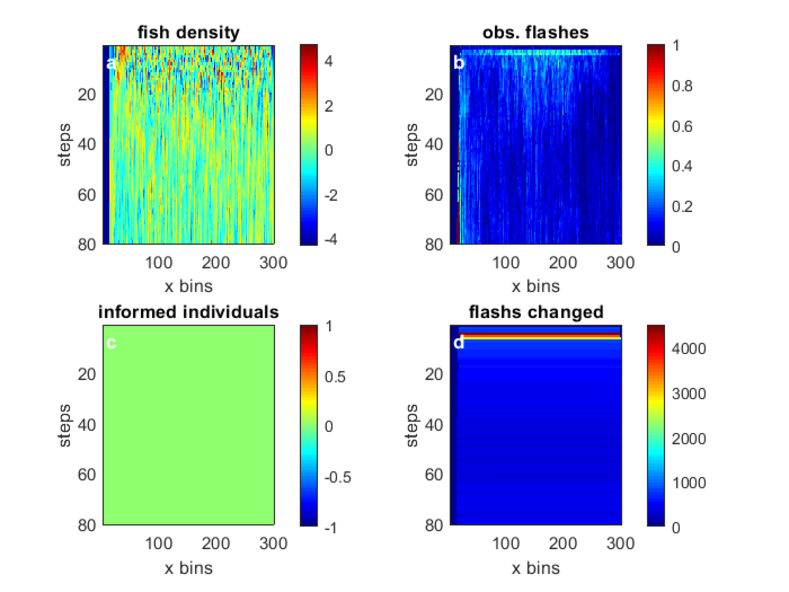

Supplement: S12 Fig — Simultaneous response of the fish to the attack of the predator. The attack is followed by an instantaneous observed flash cloud and no information transfer afterwards. Since all the fish are changing simultaneously into an emergency state, there are no detections of informed individuals. (TIF) [file pone.0289026.s013.tif]

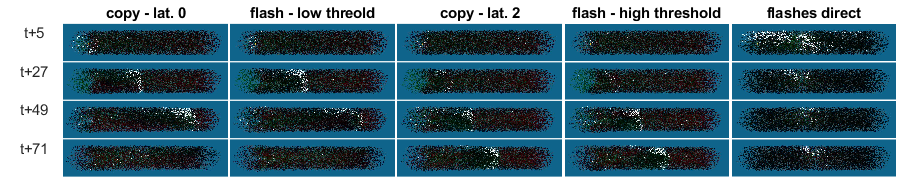

Supplement: S13 Fig — A very sensitive school behaves practically as a school with low latency, while a non-sensitive school never activates its response to the flashes and acts like a normal school with latency 2. The right column shows the direct response to flashes which results in a fast explosion all over the school. (TIF) [file pone.0289026.s014.tif]

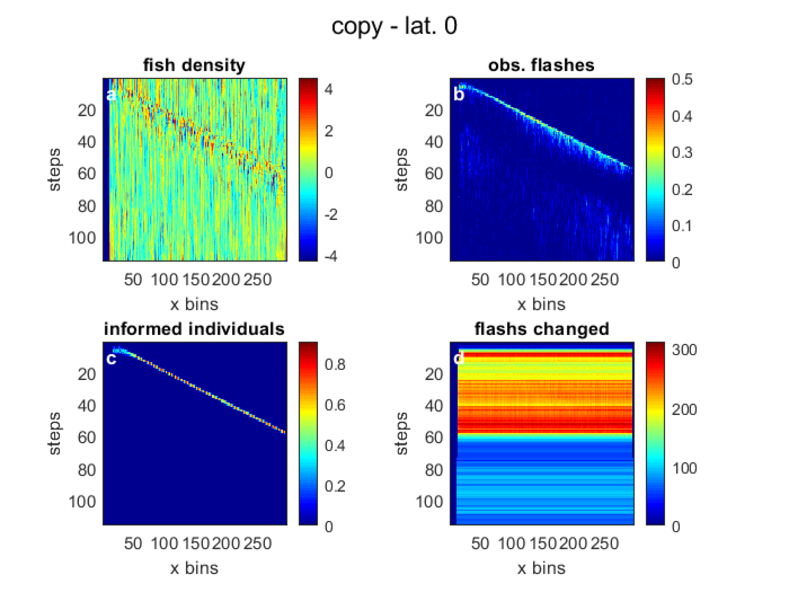

Supplement: S14 Fig — The dynamics are equivalent to those of the ‘normal’ latency but quicker. (TIF) [file pone.0289026.s015.tif]

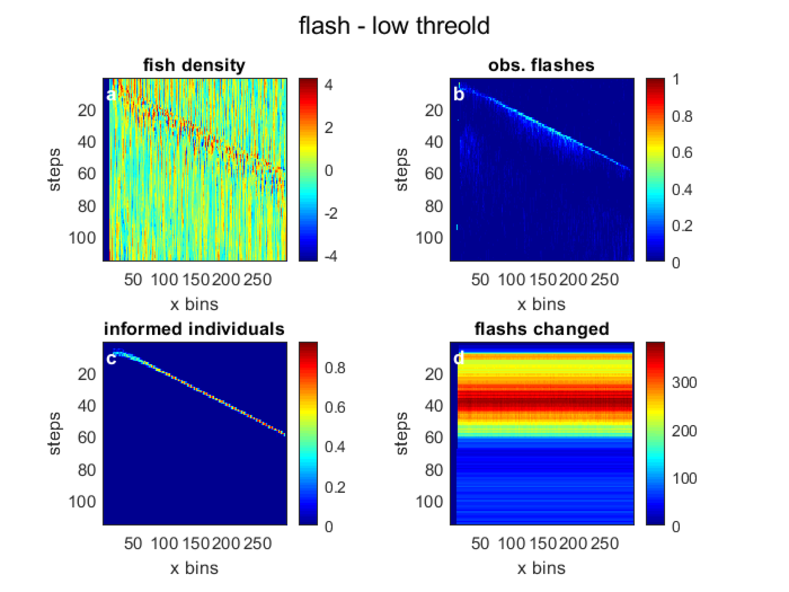

Supplement: S15 Fig — (TIF) [file pone.0289026.s016.tif]

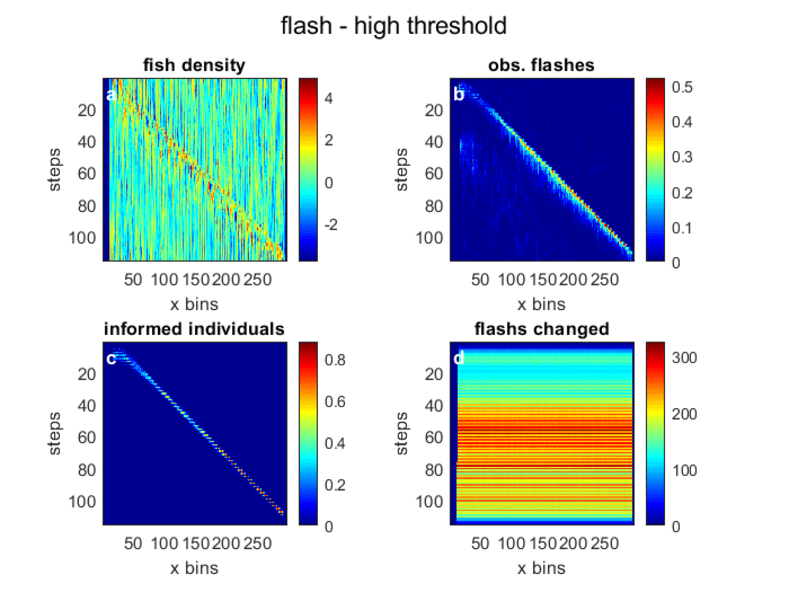

Supplement: S16 Fig — (TIF) [file pone.0289026.s017.tif]

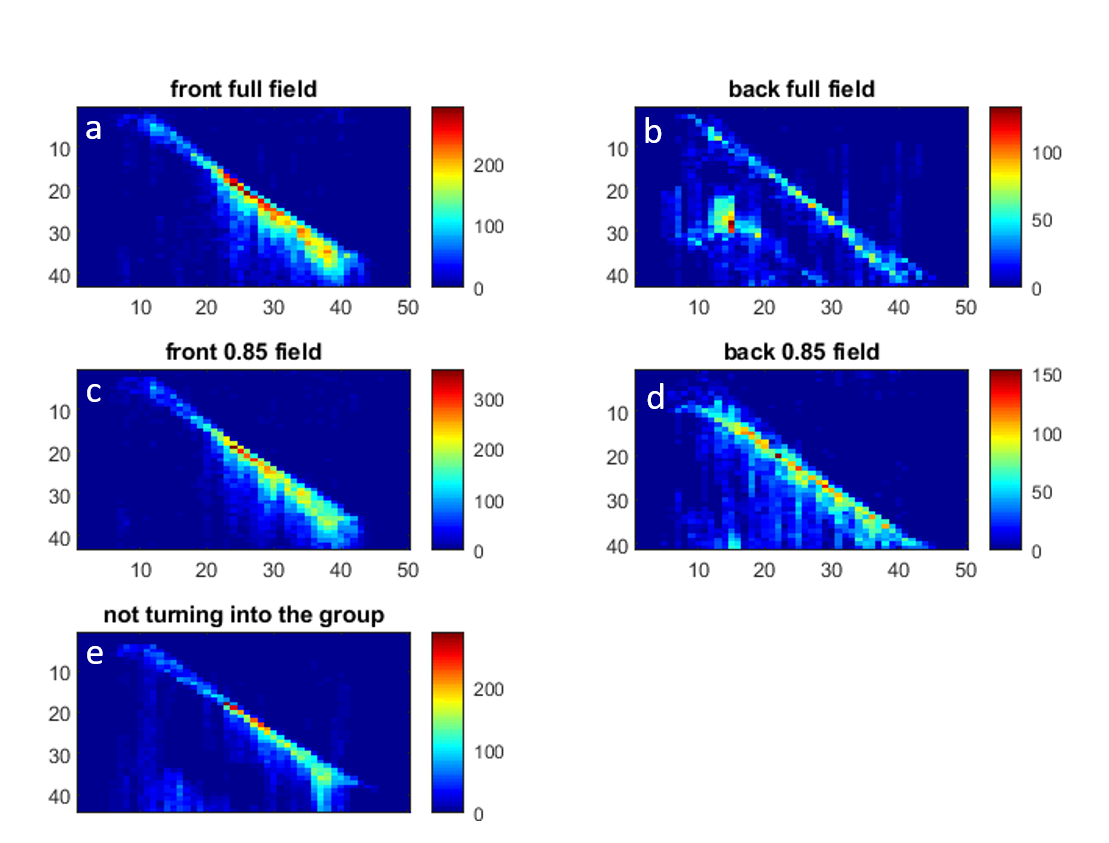

Supplement: S17 Fig — The escape response of the fish is to copy informed individuals and ignore flash signals. Plot a. Attack from the front of fish with full field of view. Plot b. Attack from the back of fish with full field of view. Plots c, d: Attack from front/back on fish with 85% field of view. Plot e. Attack from the front on fish with full viewing field while the escape response of the fish does not include turning into the group. In all plots we see that the attack leads to a flash wave in a constant speed (as in Fig 2 in the MS). The attacks from the back lead to a slightly weaker and less ordered wave but of the same speed as in the default configuration. (TIF) [file pone.0289026.s018.tif]

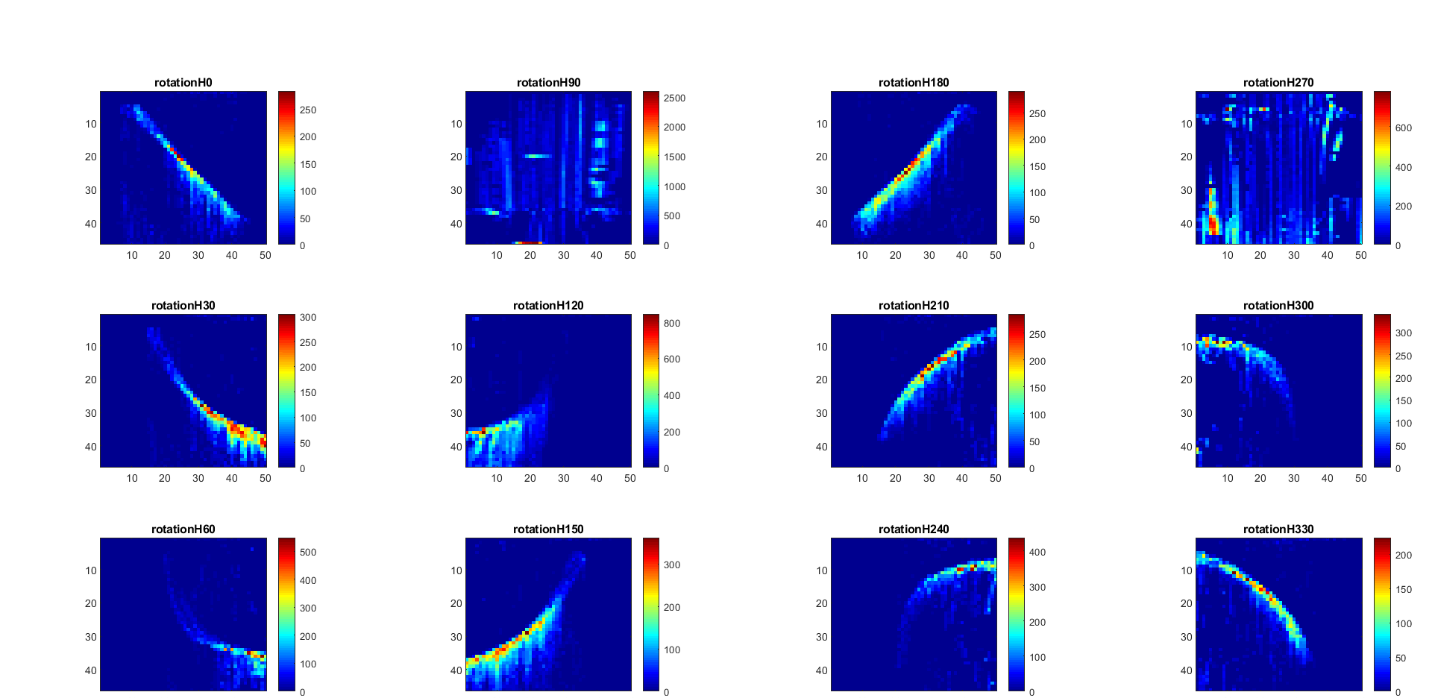

Supplement: S18 Fig — The wave pattern is clearly seen except from perpendicular angles to the wave distance (plots 90 and 270). The closer the angle to perpendicular, the less linear the visible wave speed is. (TIF) [file pone.0289026.s019.tif]

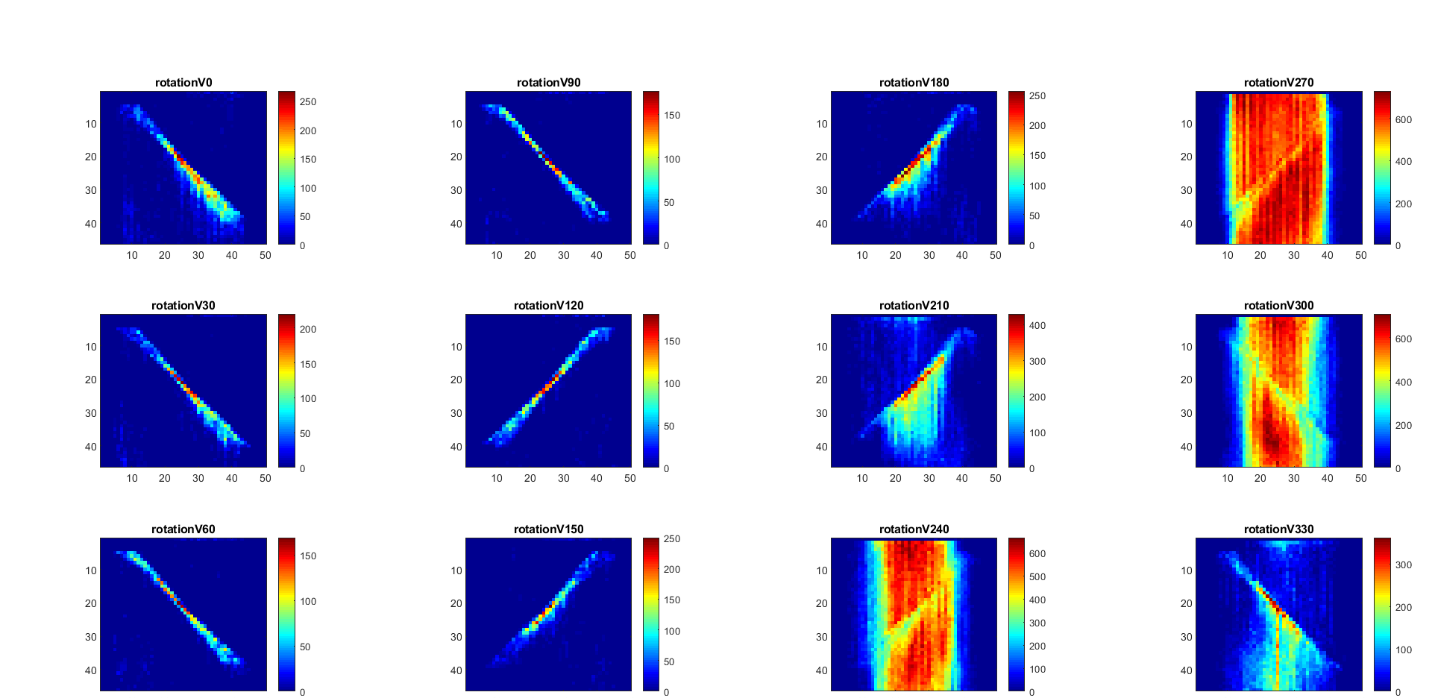

Supplement: S19 Fig — The wave pattern is visible from every angle. In particular, when looking from below (angles of 240, 270 and 300 degrees), an anti-flash wave can be seen. (TIF) [file pone.0289026.s020.tif]

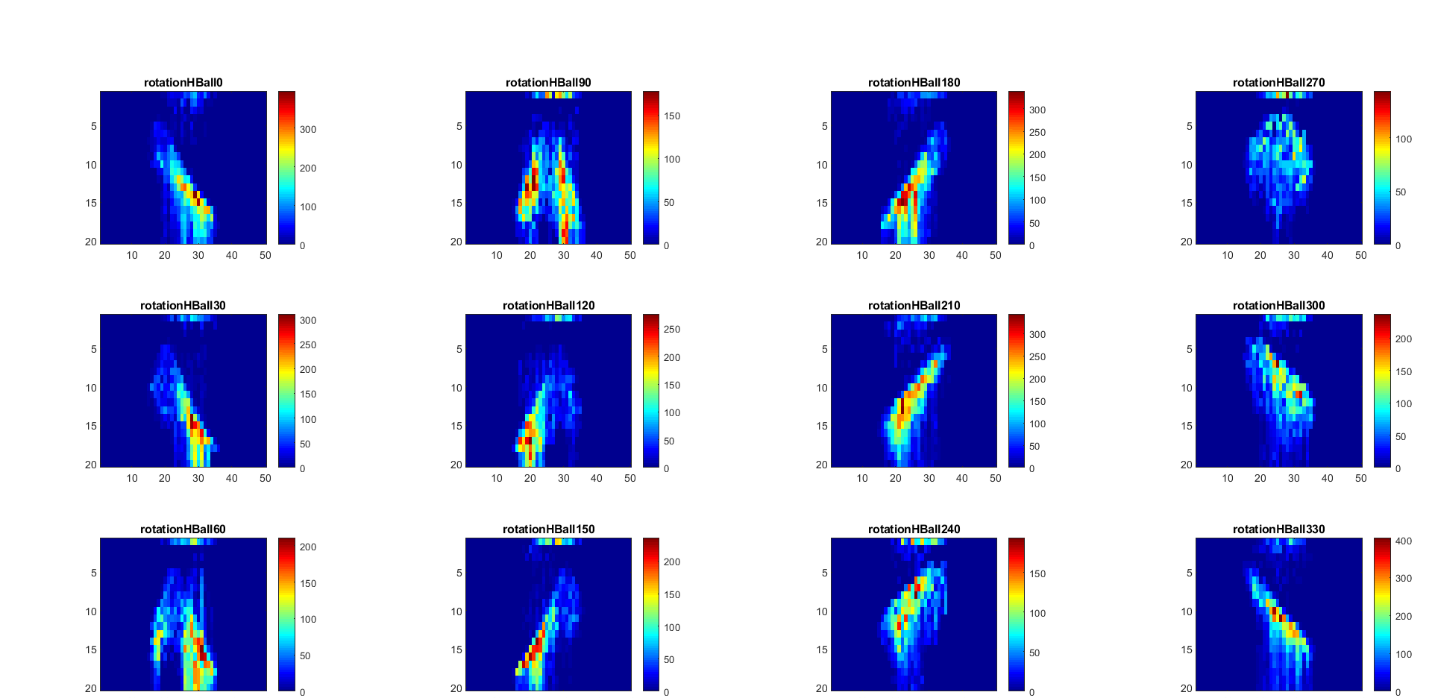

Supplement: S20 Fig — The wave pattern is clearly seen and gets distorted the more we apply perpendicular angles to the wave distance (plots 90 and 270). Even in the perpendicular angles, we can see a dynamic of monotone growth/decay of the flashes. (TIF) [file pone.0289026.s021.tif]

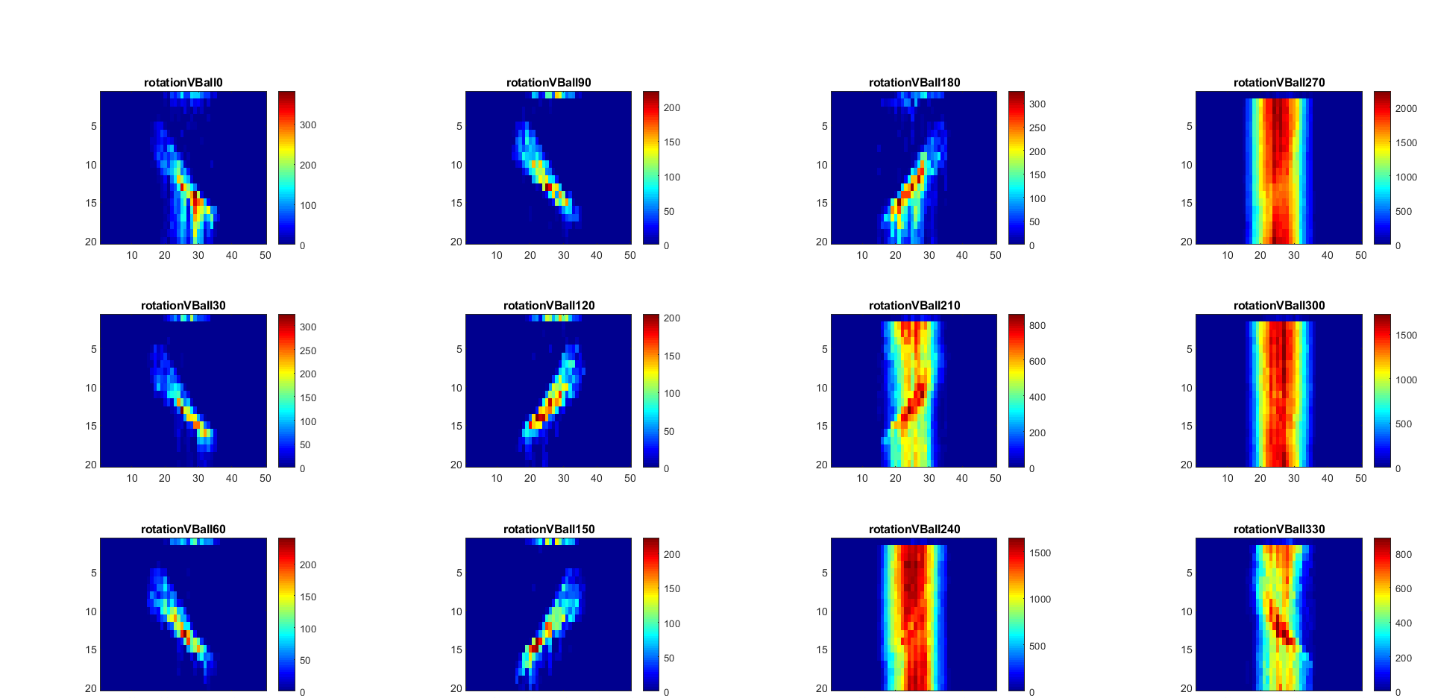

Supplement: S21 Fig — The wave pattern is visible from every angle. In particular, when looking from below (angles of 240, 270 and 300 degrees), an anti-flash wave can be seen. (TIF) [file pone.0289026.s022.tif]

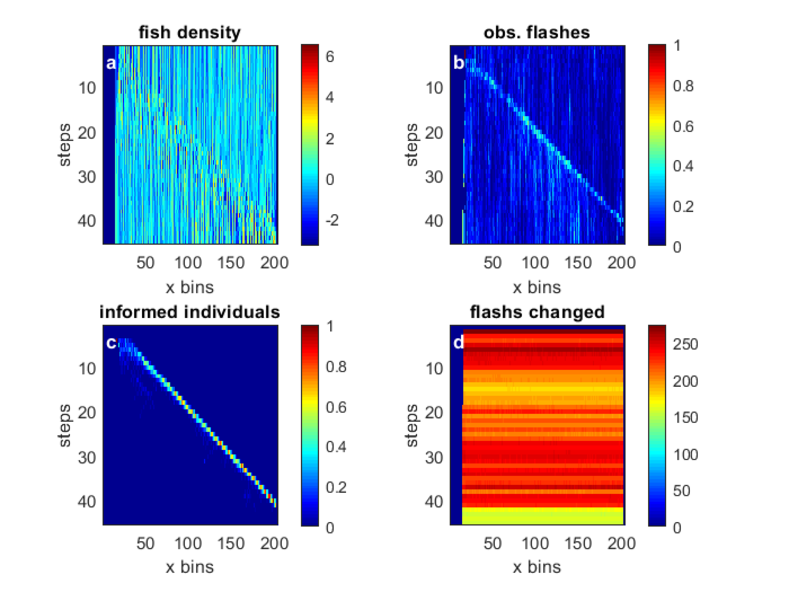

Supplement: S22 Fig — The fish density and the observed flash signals are clear but less significant than in the aligned schools. The flash-change signal cannot be seen. (TIF) [file pone.0289026.s023.tif]

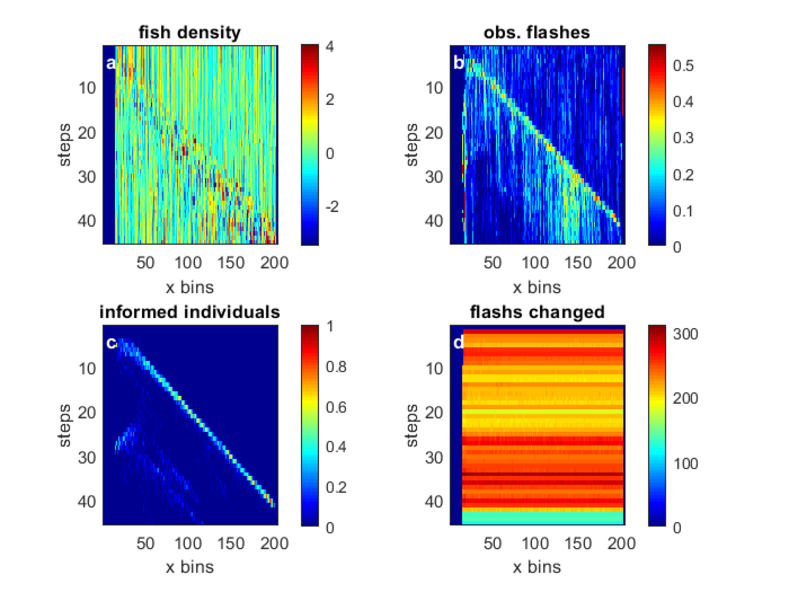

Supplement: S23 Fig — Compared to S22 Fig, the patterns of the noises slightly differ but the signals are the same. (TIF) [file pone.0289026.s024.tif]

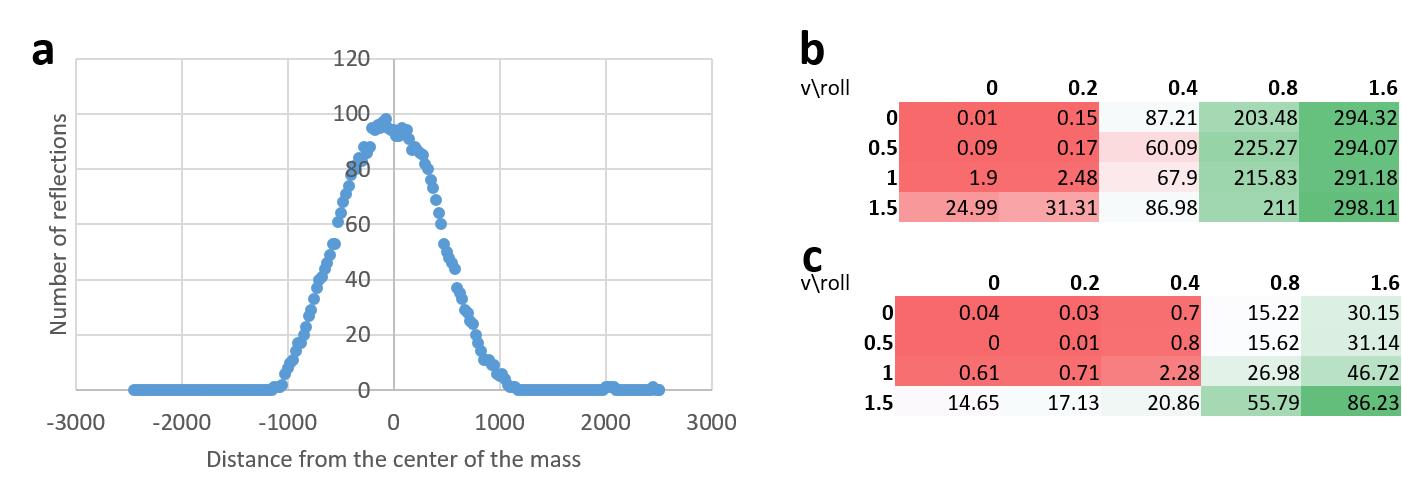

Supplement: S24 Fig — a. The number of Boids reflecting light towards an observer which moves horizontally crossing the center of the mass of an oval group of 1000 “relaxed” Boids under light distribution resembles an ideal Snel’s window. b. is a table of the average number of visible flashes towards a horizontal observer perpendicular to the school‘s swimming direction. The columns represent different noises in the roll of the fish and the rows show different noises in the direction of the velocity of the fish. The roll noises have stronger effects on the flashes. Flashes in the high velocity noise are also due to strong torsion (changes in roll) required to “correct” the noise. A similar analysis with roll always set to zero gave zero flashes in all configurations. c. shows the same scenario of plot b, except that the observer is placed facing the school’s direction. The trend of dependency on the roll-noise is preserved. The combined effect of both noises has a stronger relative influence on the number of flashes and the total numbers of flashes are lower in comparison to each one of them separately. (TIF) [file pone.0289026.s025.tif]

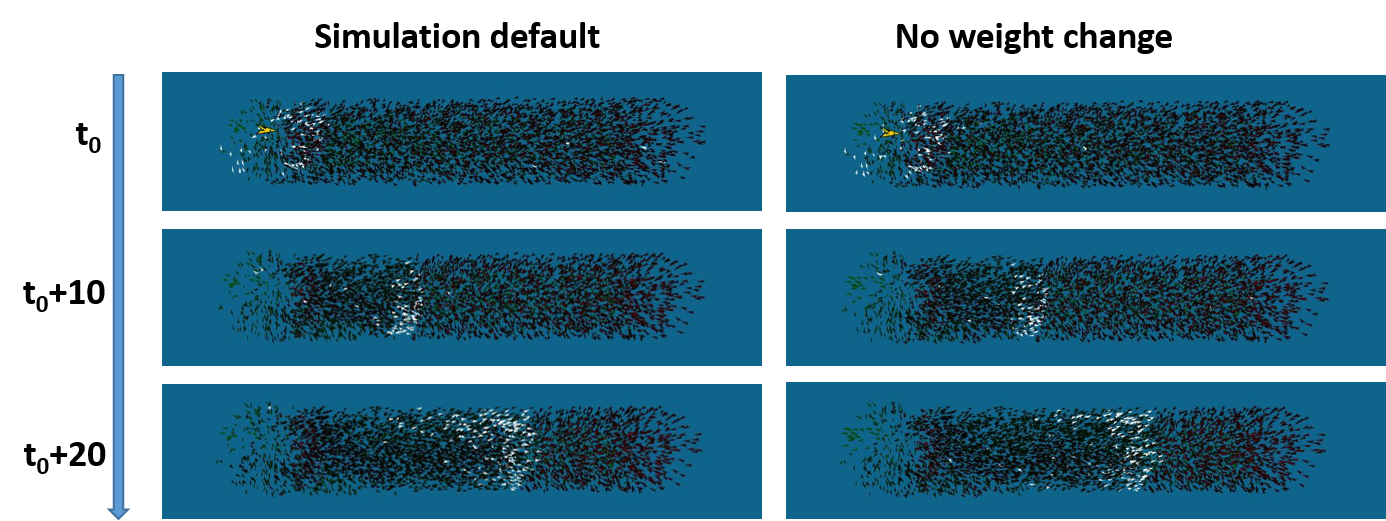

Supplement: S25 Fig — In our model, an agent in emergency mode is prioritizing cohesion and alignment over repulsion for the sake of avoiding separation from the group in strong attacks. In our tests, which were consisted of short local attacks, there is no difference in the speed of the flash wave between copy response with weights changes (left column) and copy response without weights changes (right column). (TIF) [file pone.0289026.s026.tif]
