# Supplementary material for: Schooling of light reflecting fish
Source: PLoS One. 2023 Jul 21;18(7):e0289026. doi: 10.1371/journal.pone.0289026 (PMC10361475; doi:10.1371/journal.pone.0289026)
Supplement: S1 File — (PDF) [file pone.0289026.s029.pdf]

Schooling of light reflecting fish

Supplementary Information

Assaf Pertzelan[1,2], Gil Ariel[3] and Moshe Kiflawi[1,2]

[1] *Faculty of Life Sciences, Ben Gurion University, Beer-Sheva, Israel.*

[2] *The Interuniversity Institute for Marine Sciences at Eilat (IUI), Israel.*

[3] *Department of Mathematics, Bar-Ilan University, Ramat-Gan, Israel.*

1 Model and simulation

Our model extends a highly-studied model of self-propelled particles, known as the three zones model introduced by Aoki (1982) and independently by Reynolds (1987) (see also Couzin et al. 2002; Jolles et al. 2017), in order to generate movement patterns that resemble schooling fish and birds. The simulation consists of *N* agents (here individual fish).

In the original three-zones model, each agent i at every discrete simulation time step *t* is described by its position, denoted $p_i\ (t)$ and velocity $v_i(t)$. In order to take into account predator response, we add two new additional variables that are not part of the original model, the acceleration $a_i(t)$, and a discrete internal state $s_i(t)$. The internal state is one of four options: schooling, evasive, predator-response (similar to Lee (2006)), or copy-response (similar to Sonoda et al. (2019)), as described below. Moreover, tracing the direction in which light rays reflect off the fish body requires us to also keep track of the direction corresponding to the back of the fish (the direction that regularly points upward), denoted $b_i(t)$. It is always perpendicular to the velocity.

In addition to the N fish, simulations may involve a single predator, which appears for a limited number of steps and moves along a fixed trajectory. Usually, the predator moves towards and into the school, typically faster than the agents' speed in the schooling state.

Agents dynamics:

In each step, all agents update their positions synchronously based on their current velocity,

$$p_i(t+1) = p_i(t) + v_i(t).$$

Velocity-updates depend on the current internal state $s_i(t)$ as follows (see Fig 1 in the MS).

- *Schooling*: Agent velocity is determined similar to the usual three zones rules, e.g. Couzin et al. (2002), i.e. a weighted average of attraction to all neighbours within a given distance R_o, alignment to all neighbours within a distance R_a and repulsion from neighbours within a distance R_r, such that $R_r<R_a<R_o$. See

Figure **S1** Fig for a cartoon.

To be precise, we first calculate an acceleration as follows. Define three sets of neighbouring agents within each interaction distance,

$$A_i^{\mathrm{a}}(t) = \{ j \mid |p_i(t) - p_j(t)| \le R_{\mathrm{a}} \}$$

$$A_i^{\mathrm{o}}(t) = \{ j \mid |p_i(t) - p_j(t)| \le R_{\mathrm{o}} \}$$

$$A_i^{\mathrm{r}}(t) = \{ j \mid |p_i(t) - p_j(t)| \le R_{\mathrm{r}} \}$$

where $|\cdot|$ denotes the Euclidean distance. Calculate the acceleration,

$$a_i(t) = w_{\mathrm{a}} a_i^{\mathrm{a}}(t) + w_{\mathrm{o}} a_i^{\mathrm{o}}(t) + w_{\mathrm{r}} a_i^{\mathrm{r}}(t) + \gamma_i(t),$$

where w_{a} , w_{o} and w_{r} are the weights of the attraction, alignment, and repulsion, respectively. a_i^a, a_i^o and a_i^r are the corresponding vectors, calculated as

$$a_i^{\mathrm{o}}(t) = \frac{1}{|A_i^{\mathrm{o}}(t)|} \sum_{j \in A_i^{\mathrm{o}}(t)} p_j(t) - p_i(t)$$

$$a_i^{\mathrm{a}}(t) = \frac{1}{|A_i^{\mathrm{a}}(t)|} \sum_{j \in A_i^{\mathrm{a}}(t)} v_j(t)$$

$$a_i^{\mathrm{r}}(t) = \frac{1}{|A_i^{\mathrm{r}}(t)|} \sum_{j \in A_i^{\mathrm{r}}(t)} \frac{p_j(t) - p_i(t)}{|(p_j(t) - p_i(t)|}$$

where $|A|$ denotes the number of elements in a set A. Each of the accelerations a_i^{a}, a_i^{o} and a_i^{r} has a maximum length $a_{\max}$, for example, if $|a_i^{\mathrm{o}}| > a_{\max}$ then $a_i^{\mathrm{o}} = a_i^{\mathrm{o}} a_{\max}/|a_i^{\mathrm{o}}|$, and similarly for a_i^{a} and a_i^{r}.

$\gamma_i(t)$ describes several adjustments to the traditional motion rules as follows,

$$\gamma_i(t) = w_{\mathrm{m}} m + w_{\mathrm{d}} a_i^{\mathrm{d}}(t) + w_{\mathrm{w}} a_i^{\mathrm{w}}(t) + \psi(t),$$

where

- The vector m is a constant that describes a preferred direction, i.e. an agent "wants" to migrate in the direction *m* (see Table 1).
- $a_i^{\mathrm{d}}(t)$ is vector that moves the agents towards a desired water depth (value on the *z*-axis, arbitrarily set to zero),

  $$a_i^{\mathrm{d}}(t) = -p_i(t).z|p_i(t).z|i_3,$$

  where $i_3 = (0,0,1)$ and $[\cdot].z$ is the z component of the vector. See Figure **S2** Fig for an example for the trajectory of a single agent, correcting its depth using this approach.
- a_i^{w} is an attraction to a desired value on the *x*-axis calculated in the same manner as a_i^{d}.
- $\psi(t)$ is a Gaussian noise term with zero mean and covariance matrix $\sigma^2 I_3$. Here, I_3 is the 3x3 identity matrix.

Finally, the new velocity is taken to be,

$$v_i(t+1) = Iv_i(t) + a_i(t),$$

where I is an inertia parameter (default value 0.95), qualitatively modeling the friction of water.

- *Evasive*: If an agent in the schooling state gains a speed that is higher than the schooling speed limit $|v_i(t)| > s_{\max}^{\text{schooling}}$ (for example, because it encountered a predator or an informed individual − see copy-response section), it transitions to an evasive state. In this state, the update rules for the velocity are similar to the rules while schooling, but with different weights that give higher priority to cohesion and alignment over repulsion. The evasive state lasts until the fish slows down by friction and by averaging out their response with their schooling neighbors.

$$a_i(t) = w_{\text{oe}} a_i^{\text{o}}(t) + w_{\text{ae}} a_i^{\text{a}}(t) + w_{\text{re}} a_i^{\text{r}}(t) + \gamma_i(t),$$

- *direct-only*: If an agent in the schooling or evasive states encounters the predator (i.e. the predator is within a distance R_p), then the agent transitions into the predator-response state. In this state, agents turn towards the opposite direction of the predator and start swimming at speed $s_{\max}^{\text{evasive}}$,

$$v_i(t) = s_{\max}^{\text{evasive}} \frac{p_i(t) - p_{\text{predator}}(t)}{|p_i(t) - p_{\text{predator}}(t)|}.$$

- *direct-and-copy*: A copy-response behaviour describes a *schooling* agent that is not within sight range of a predator (R_p), not in the middle of a sharp change of its trajectory (the angle between consecutive velocity vectors < 45°), and not in the evasive state, but has a neighboring agent (within radius R_i) which is making a sharp turn and swimming fast (above a threshold) will

transition into the copy-response state. To be precise, an informed individual is a neighbor within a range R_i that made the sharpest turn, given that the turning angle is larger than a threshold (~80° in our implementation) and the neighbor's speed is high enough (0.5 of the maximum escape speed $s_{\max}^{\text{evasive}}$). In this state, agents copy the velocity of the informed neighbor. In (the unlikely) case that there are more than one "sharpest turn" neighbors one of them is chosen arbitrarily. The rule for the velocity update is given by

$$v_i(t + latency) = v_{\text{infInd}_\text{i}}(t - 1),$$

where,

$$infInd_i(t) = \min_{j \in A_i^i(\text{t}) \; \& \; |v_j| > s_{\max}^{\text{evasive}}/2} v_j(t) \cdot v_j(t-1) \; .$$

Here, *latency* is a parameter. This parameter is the only one which is modified by response to flashes, as explained below.

Roll angles:

As explained above, in order to track the angle in which incident light beams are reflected off agents, one needs to model the dynamics of a direction normal to the velocity, corresponding to the back of the fish. We denote this direction as $b_i(t)$. In simulations, $b_i(t)$ is given by

$$b_i(t) = w_{tor} tor_i(t) + w_{ra} b_i^{ra}(t) + w_{bt} i_3 + \psi_\text{r},$$

where $tor_i(t)$ is the torsion of the trajectory curve of the agent,

$$tor_i(t) = b_i(t-1) + \; v_i(t) - 2v_i(t-1) + v_i(t-2),$$

and $b_i^{\text{ra}}(t)$ is a roll-alignment motion rule

$$b_i^{\mathrm{ra}}(t) = \frac{1}{|A_i^{\mathrm{ra}}(t)|} \sum_{j \in A_i^{\mathrm{ra}}(t)} b_j(t) \,.$$

Similar to previous notation, $A_i^{\mathrm{ra}}(t)$ is the set of neighbors of fish i up to a distance R_{ra} and $\psi_{\mathrm{r}}(t)$ is added Gaussian noise with variance $\sigma_{\mathrm{r}}^2 I_3$.

Optics: See Fig in the MS.

We assume a light source that consists of two components: 1. we define a light direction vector $ld = -i_3 = (0,0,-1)$ and 2. an angle θ representing a uniform light distribution around the light direction. The agents are considered as 2-sided planar mirrors whose normal direction $n_i(t)$ is perpendicular to the velocity and back vectors,

$$n_i(t) = \frac{v_i(t) \times b_i(t)}{|v_i(t) \times b_i(t)|}.$$

In order to determine if agent i reflects light towards position x in a given time t, we define a vector

$$ray_i(t) = \frac{(p_i(t) - ld}{|(p_i(t) - ld|},$$

in the direction of $p_i(t) - ld$.

Then, we define the reflected vector of $ray_i(t)$ from the plane represented by the normal $n_i(t)$,

$$ref_i(t) = ray_i(t) - 2n_i(t)\big(ray_i(t) \cdot n_i(t)\big),$$

and another vector pointing from the agent position towards x,

$$obs_i^x(t) = \frac{x - p_i(t)}{|x - p_i(t)|}.$$

If the angle between $ref_i(t)$ and the vector $obs_i^x(t)$ is smaller than θ, then the agent reflects light towards x. Denote by $f_i^x(t)$ a Boolean variable describing if agent *i* reflects light towards position x,

$$f_i^x(t) = \begin{cases} T & \text{acos}(obs_i^x(t) \cdot \text{ref}_i(t)) > \theta \\ F & \text{otherwise} \end{cases}.$$

Since the model is discrete, it is possible that an agent will not reflect light both in time t and in time $t + 1$, but the trajectory between the two times will cross a point in which it will flash. To test whether an agent was flashing between the steps, we take

$$refn_i(t) = ref_i(t) \times \text{ref}_\text{i}(t - 1)$$

as the plane normal defined by the reflected rays in times t and $t - 1$, and check the angle between this plane to $obs_i^x(t)$. If the projection is falling on the arc drawn by the trajectory between the two reflection rays, and the angle is smaller than θ, the fish is flashing. We consider the agent as flashing in time t in such case. This continuous flashing (the flashing between steps) is given by

$$fc_i^x(t) = f_i^x \ or \ 1 - obs_i^x(t) \cdot \ refn_i(t) < \text{threshold}$$

Detection of the flashes by the agents:

We add a new assumption that agents can detect flashes and that the accumulative effect of many flashes can affect the dynamics. We assume that if an agent sees many new light reflections within a single step, or many existing reflections are turned off, it assumes that these changes indicate instability in the motion of the group. As a precaution (a *flash-response* state) they lower the latency (so it will respond faster). In other words, the effect of flashes only changes the latency in the transition from schooling to evasive-copy behaviour.

The way we quantify the total number of changes in the flash is by summing the number of agents that changed the value of $fc_j^{p_i}$ between time t to time $t+1$. We defined a set

$$A_i^{\mathrm{fc}}(t) = \left\{j | fc_j^{p_i}(t) \oplus fc_j^{p_i}(t+1)\right\},$$

where $\oplus$ denotes exclusive or. Therefore, $A_i^{\mathrm{fc}}(t)$ is the set of agents which flashed towards agent *i* at time *t*, but not at time *t*+1, or the other way around - flashed towards agent *i* at time *t*+1, but not at time *t* . The flash change measure is simply $fc_i(t) = |A_i^{\mathrm{fc}}(t)|$. We defined a flash-change threshold fct (parameter) and implemented two possible responses to this flash change, both options assume that the fish is in schooling state:

1. *Flash-direct*:

$$v_i(t+1) = \frac{|v_i(t)|}{fc_i(t)} \sum_{j \in A_i^{\mathrm{fc}}(t)} p_i(t) - p_j(t),$$

i.e. the agent turns away from the center of mass of the changed flashes, keeping its speed from time t. The idea behind it is that the center of mass of the flash changes may indicate the location of the cause of the disturbance.

2. *Flash latency*: An agent that is exposed to above-threshold flash changes, changes its latency to one. The idea is that the flash changes inform the fish that something may be wrong without giving precise information. Because of that, the response of the fish is to be ready to react faster and anticipate more specific information.

Figure S1. The relative dimensions of the copy-response zone and the three social zones that give rise to schooling state. The agent (the black fish in the middle) modifies its motion based on its neighbors' velocities and locations within the Cohesion zone (the agent is attracted towards the center of the mass of the neighbors' position within this zone, which is typically the largest). The Alignment zone (the agent adjusts its direction towards the average direction of its neighbors within this zone) and the Repulsion zone (the agent tries to get away of the agents in this zone by swimming away of their center of mass weighted by their relative distance from the agents' position. This zone, out of all zones, is typically the smallest and of the highest weight). Fish within the copy zone look for informed neighbors, whose swimming speed and direction they copy. Our default for this zone is the same as the alignment, assuming that from this distance the fish can clearly see changes in orientation.

Figure S2. Example for the trajectory of a single agent, correcting its depth.

Table S1. Parameters table for the examined scenarios.

Parameter(s)	Default value(s)	Comments
$\boldsymbol{N}$	15000	School size. Typically 1000 or 15000
$\boldsymbol{I}$	0.95	Inertia
$\boldsymbol{RC}, \boldsymbol{RA}$, $\boldsymbol{RR}, \boldsymbol{RRA}$, $\boldsymbol{RP}, \boldsymbol{RI}$ **(mm)**	1000,600,400,500, 1000, 600	Radii of the spheres where the neighbouring agents are used for cohesion, alignment, repulsion, and alignment-roll, predator detection, informed individual detection. The parameters were chosen based on realistic estimation of distance between individuals which is approximately resembles the repulsion distance. Empirically number of neighbours in sphere are *RC*~=250, *RA*~=65, *RR*~=20,*RRA*~=40,*RP*~=250,*RI*~=65
$\boldsymbol{w_c}, \boldsymbol{w_a}, \boldsymbol{w_r}, \boldsymbol{w_{ra}}$	1.3,1.5,2.5,0.1	Weights of the rules for cohesion, alignment, repulsion, and alignment-roll in schooling state
$\boldsymbol{w_{ce}}, \boldsymbol{w_{ae}}, \boldsymbol{w_{re}}$	4,4,1	Weights of the rules for cohesion, alignment, and repulsion in evasive state. Those values were chosen to ensure the cohesion of the group
$\boldsymbol{w_d}, \boldsymbol{w_w}, \boldsymbol{w_m}$	0.1,0.1,0.1	Weights for correction towards depth, width, and migration; where applied on the weighted acceleration vector after defining it by the motion rules
$\boldsymbol{c_{depth}}$**(mm)**	1000	
$\boldsymbol{m}$**(mm/step)**	(0,0,0.1)	The migration vector

a_{max}**(mm/step)**	0.1	
$s_{max}^{schooling}$ **(mm/step)**	6	
$s_{max}^{evasive}$ **(mm/step)**	30	Speed of escape from a predator
σ, σ_r	0.5,0.2	Standard deviations of the noises of the velocity and the back vector.
$latency$ **(steps)**	3	1 is often used as well (when response to flashes was not relevant)
ld, θ	(0,0,-1), 46^0	Light direction and distribution, using the ideal Snell's window
fct	400	Threshold of flash changes. 175 to the 5k scenarios

2 Sensitivity of the wave speed to parameters

Figure S3. Wave speed as a function of parameters: a. latency, b. escape speed, c. response distances of all the motion rules (see default values in S1 Table), d. the distance at which the fish applies the copy-response.

Figure S3.2 Sensitivity to rules weights. In our model, an agent in emergency mode is prioritizing cohesion and alignment over repulsion for the sake of avoiding separation from the group in strong attacks. In our tests, which were consisted of short local attacks, there is no difference in the speed of the flash wave between copy response with weights changes (left column) and copy response without weights changes (right column).

3 Example simulated snapshot sequence

Figure S4 shows frames from two samples: the flash signature caused by response to an attack on a cylindrical and a spherical school. The flash signatures of the two samples are clearly not identical, but it is hard to tell intuitively which of the differences are due to random factors in the sample (e.g. the location of the observer or noise in the orientation of the fish), and which are actually features characterizing the two different school shapes. These samples are used for training and testing our classifier.

Figure S4. A comparison between the sequences of flashes generated by a simulated attacks on a cylindrical (top) and a spherical (bottom) school. The attack came from the opposite direction to the school movement. The observer does not 'know' its position relative to the school (based on the flashes only) on the horizontal plane, and the center of the field of view of the observer is not fixed. Some of the flashes are due to random movement of the fish around their roll axis, while other are due to evasive or copy responses.

4 Classification model

Our model. The model was written in Python using Keras and was trained and tested on the platform of Google Colabs. Our model consists of a ConvLSTM2D layer which expects tagged sequences of 90x90 grayscale images. A dropout of 0.2 was added to this layer. This layer is made of 64 convLSTM cells each and applies 5x5 convolution filters as their gates and input/output. The output of this layer is transformed to be one-dimensional and is transferred to a fully connected layer (dropout: 0.3). The nodes in this layer are Rectified linear units (ReLU). Fully connected ReLU layers are often used as default layers because of performance considerations during training [33]. This ReLU layer is fully connected to our output layer, which consists of nodes at the same number of classes in our dataset that apply softMax on the input. The softMax function is normalizing the inputs into probabilities for each class with amplification of the probabilities of the higher input values. See SI for the code and its description. Training: the data was divided approximately 4/5 for training (~120 samples per class in our case) by 40 epochs of batch size 8. The remaining 1/5 of the dataset (~30 samples per class in our case) was left for testing. A patience of 7 was added to avoid overfitting.

Evaluation of the model. To analyze the quality of a trained model with the test datasets we used confusion matrices that compare the frequency of true and the predicted classes (~30 samples per class). From the confusion matrices we calculated the accuracy, precision, recall and f1-scores.

Random noise. To test the robustness of the classification to missing information, we added three (optional) uncertainty parameters (S5 Fig). These parameters were set to reflect information that is not available to the observer in realistic situations: a. *roll-noise*: The degree of random roll of the fish around its head-tail axis, b. *lookat-moves*: The observer's position could stay relatively

fixed but the direction of the center of its field of view may move (for example, near a wavy sea surface), and c. *observer-orientation*: An observer can tell where it stands in relation to the school on the vertical plane (if it is looking up or down) but not in the horizontal plane.

Figure S5. Uuncertainty parameters. a. noise in the motion of the fish around the roll axis. b. noise in the look-at point of the observer. c. noise in the location of the observer on the horizontal plane around the school.

5 Full results for the information content of shimmering waves

In this section we present the full results of the three models, each with and without the uncertainties (i.e. with or without roll-noise, lookat-moves, and observer-orientation. See ‘*Partial information and noise*’ section in the MS for details). For each model we present ‘classification report (details in the figure below) as well as the confusion matrix.

5.1 Table of terms

Table S2 measurements of the DNN

Term	Explanation
**Accuracy**	(True negatives + true positives)/(total number of samples)
**Precision**	True positives/(true positives+false positives)
**Recall**	True positives/(true positives+false negatives)
**F1-score**	2*precision*recall/(precision+recall)

5.2 The classification report structure

Figure S6. The classification_report output structure. The names of the classes are presented in the top row by the order they appear in the table. For each class we measure the precision, recall, and f1-score. The total accuracy is being calculated for the entire model. Additionally, the *support* column presents the number of samples we used for testing each class. The last two rows present the weighted, and the non-weighted averages for precision, recall and f-score for the entire model. Since our dataset is balanced, the averaged f1-score is similar to the total accuracy, and there is not much difference between the weighted and the non-weighed averages.

5.3 Attack direction without uncertainty parameters

```
classification report for classes: ['noAttack', 'side', 'bottom', 'rear',
'top', 'front']
              precision    recall  f1-score   support


    noAttack       1.00      1.00      1.00        25
        side       0.77      0.87      0.82        31
      bottom       0.90      0.67      0.77        27
        rear       0.77      0.88      0.82        34
         top       0.90      0.81      0.85        32
       front       0.97      1.00      0.98        31
```

accuracy			0.87	180
macro avg	0.88	0.87	0.87	180
weighted avg	0.88	0.87	0.87	180

Figure S7. **Confusion matrix for attack direction**, without uncertainty parameters.

5.4 Attack direction with uncertainty parameters

classification report for classes: ['noAttack', 'side', 'bottom', 'rear', 'top', 'front']

	precision	recall	f1-score	support
noAttack	0.96	0.88	0.92	25
side	0.70	0.68	0.69	28
bottom	0.68	0.88	0.77	32
rear	0.60	0.67	0.63	27
top	0.85	0.58	0.69	38

front	0.74	0.81	0.77	31
accuracy			0.74	181
macro avg	0.75	0.75	0.74	181
weighted avg	0.75	0.74	0.74	181

- The confusion matrix is presented in the MS in Fig 7.

5.5 School shape without uncertainty parameters

classification report for classes: ['cylinder', 'pancake', 'ball']

	precision	recall	f1-score	support
cylinder	1.00	1.00	1.00	28
pancake	1.00	1.00	1.00	31
ball	1.00	1.00	1.00	31
accuracy			1.00	90
macro avg	1.00	1.00	1.00	90
weighted avg	1.00	1.00	1.00	90

Figure S8. Confusion matrix for school shape, without uncertainty parameters.

5.6 School shape with uncertainty parameters

```
classification report for classes: ['cylinder', 'pancake', 'ball']
              precision    recall  f1-score   support

    cylinder       0.87      0.96      0.92        28
     pancake       0.96      0.87      0.92        31
        ball       1.00      1.00      1.00        31

    accuracy                           0.94        90
   macro avg       0.95      0.95      0.94        90
weighted avg       0.95      0.94      0.94        90
```

- The confusion matrix is presented in the MS in Fig 8.

5.7 Fish response without uncertainty parameters

```
classification report for classes: ['dirOnly', 'flashesDir', 'flashesLat',
'dirAndCopy']
              precision    recall  f1-score   support

     dirOnly       1.00      1.00      1.00        28
  flashesDir       1.00      1.00      1.00        28
```

```
  flashesLat       1.00      1.00      1.00        36
  dirAndCopy       1.00      1.00      1.00        28

    accuracy                           1.00       120
   macro avg       1.00      1.00      1.00       120
weighted avg       1.00      1.00      1.00       120

-------------------------------------------------------------
confusion_matrix for classes: ['dirOnly', 'flashesDir', 'flashesLat',
'dirAndCopy']
[[28  0  0  0]
 [ 0 28  0  0]
 [ 0  0 36  0]
 [ 0  0  0 28]]
zehu
```

Figure S9. Confusion matrix for fish response, without uncertainty parameters.

5.8 Fish response with uncertainty parameters

```
classification report for classes: ['dirOnly', 'flashesDir', 'flashesLat',
'dirAndCopy']
              precision    recall  f1-score   support

     dirOnly       0.85      0.82      0.84        28
  flashesDir       0.83      0.86      0.84        28
  flashesLat       0.68      0.69      0.68        36
```

dirAndCopy	0.59	0.57	0.58	28
accuracy			0.73	120
macro avg	0.74	0.74	0.74	120
weighted avg	0.73	0.73	0.73	120

- The confusion matrix is presented in the MS in Fig 11.

6 Rationale of the selected models for differentiation

1. Attack direction: In case of a predator attack. We show that the flash signature of the school contains enough information to indicate that an attack has happened. We also show that it is possible to detect the direction of the attack. This indicates that the flashing signature could be a source of information in predator-prey dynamics.

2. School shape: We show that light pattern can be used to distinguish between school shapes. This proof-of-concept suggests that light patterns can be used to study the global, coarse-grained topology of the school (e.g. also density and orientation). To the best of our knowledge, the only current method to infer the shape and structure of large schools is based on the use of Eco sounders [1].

3. Fish response: We show that light patterns could be used to distinguish among the four predator-evasion strategies (direct-only, direct-and-copy, flash-direct, and flash-latency). Success with this task will provide proof-of-concept that our method could aid in identifying the rules of collective-motion during predator attack. In particular, it will provide indication of whether or not the fish themselves are using the flashes perceived from within the school to speed up the propagation of information.

7 Flash occlusions

The model described above does not take into account occlusions, i.e., cases in which rays are blocked by other fish. Here, we study a modified model in which occlusions are not ignored, demonstrating the results are qualitatively the same. To be precise, results with and without occlusions are quantitatively the same up to a scaling factor.

Occlusions were not considered in the measurement of the flash changes. We can get them graphically for a single (or a few) point(s), but calculating them for all fish would exceed a realistic workload. In S10 Fig we placed the camera at the back of the group of fish, let the predator attack, and for each pair of subsequent frames count the pixels with flash changes. I did this once with occlusions (painting the black fish) and once without. The model is of a copy behavior type without response to flashes. The occlusion version and the non-occlusion version are strongly correlated. The area itself is also not ideal measurement since the fish may flash “in between” frames while its cross-section is different than the one presented.

Figure S10. The effect of flash change with and without occlusions from a representative point at the back of the school. a. The flashes the observer sees on time t+20 with occlusions. b. The flashes the observer sees without occlusions. c. The “real” image the observer sees. Note that the red and the green are visual aids for us indicating whether the fish turning towards the observer or away of it. They are not “seen” by the observer. Also, the image here is not limited by the distance the observer “sees” when it calculates its Boids rules. d. Correlation between the changes in total area of the flashes with and without occlusions in

the different steps. Each plot (and dataset) was calculated on a different run but is replicable. It is also important to note that in this simulation the fish are two-dimensional and in real three dimensional schools the effect of occlusion may be stronger and depend in the school density.

8 Results with the direct-only model

Here, we report additional simulation results establishing the absence of density or flash waves in the direct-only model without the copy response).

Figure S3. The four measurements for the *direct-only* model. The only pattern that could be clearly detected is the local predation event on the left side of the school at the beginning of the scenario. On plot d we can see that this event generated a weak flash signal towards the entire school.

9 Results for the flash-direct model

We tested the option of simultaneous response (*flash-direct*) by running the *direct-only* model while the predator detection distance was set to practically infinite. This resulted in all of the individuals instantaneously and directly responding to the appearing predator. Hence, a collective single "flash cloud" that decays immediately is being produced. S12 Fig shows that except for a short abrupt increase in the flash signal at the moment of the attack (similarly to the local flash

cloud that is created in the *direct-only* model), no clear flash pattern was created. This instantaneous cloud is partially a feature of the granularity of the simulation. In the simulation, a U-turn of an individual takes a single step and the individual is flashing if it was flashing at any point between its starting and ending of the U-turn. The observed simulated instantaneous dynamic is correct when the school orientation or the light field are 'noisy enough' and the observer is 'far enough'. If the observer is close to the school and the conditions are 'smooth' then other dynamics may appear, typically in a form of a quick wave that may be confused with the copy or the flash-responses.

Figure S4. *Flash-direct*. Simultaneous response of the fish to the attack of the predator. The attack is followed by an instantaneous observed flash cloud and no information transfer afterwards. Since all the fish are changing simultaneously into an emergency state, there are no detections of informed individuals.

10 Sensitivity of copy and flash responses

Flash waves can be reproduced in both the direct-and-copy and flash-latency models. Here, we compare the two model as well as the flash-direct model, in particular the dependence on parameters.

Figure S5. Comparison of low and high thresholds flash-response models to schools with no flash-response and “normal” (2) or fast (0) latencies. A very sensitive school behaves practically as a school with low latency, while a non-sensitive school never activates its response to the flashes and acts like a normal school with latency 2. The right column shows the direct response to flashes which results in a fast explosion all over the school.

Figure S6. The 4 measures for short latency, as the one the fish are switching to when getting into emergency mode. The dynamics are equivalent to those of the ‘normal’ latency but quicker.

Figure S7. When the school is very sensitive to changes in flashes, the latency is practically always 0.

Figure S8. Low sensitivity leaves the school as if it would practically not respond to flashes.

11Robustness of the wave

In the MS we tested the waves on groups of a size of 15k where the Boids had a full viewing field and the attack came from the front. The escape response of the Boids were directed into the group and then away of the predator. We tested alternative scenarios where the attack came from the back and the Boids had a partial viewing field (a blind spot 30% of the back of the fish (Rountree and Sedberry 2009)). We also tested a scenario where the fish escape directly away of the predator without turning into the group first. In all cases we found that the wave exists and its speed is essentially the same in all configurations.

We also tested different angles. In order to do so, we let the observer to “surround” an attack scenario on a 5 k cylindrical school both horizontally (S17 Fig) and vertically (S18 Fig) and recorded the waves in 10° differences. We found that except for “front view” of the school perpendicular to the wave direction, the wave pattern was detected in all angles. Interestingly, in the ‘bottom view’ (S19 Fig plots 240, 270, and 300 degrees) where all the fish are permanently reflecting light, we found an anti-flash wave.

Another interesting insight is that the wave speed was not constant from a diagonal horizontal view (e. g. angle 30 in S19 Fig). This is due to a difference in the distance between the close and the far part of the school. Checking the same angles on ball-shaped schools (S20 and S21 Fig) moderated this effect and instead of accelerating wave the effect is of smear.

Figure S9. Plots showing the visible flashes for 5 different attack scenarios on 5,000 fish. The escape response of the fish is to copy informed individuals and ignore flash signals. Plot a. Attack from the front of fish with full field of view. Plot b. Attack from the back of fish with full field of view. Plots c, d: Attack from front/back on fish with 85% field of view. Plot e. Attack from the front on fish with full viewing field while the escape response of the fish does not include turning into the group. In all plots we see that the attack leads to a flash wave in a constant speed (as in fig 2 in the MS). The attacks from the back lead to a slightly weaker and less ordered wave but of the same speed as in the default configuration.

Figure S10. A flash wave as recorded from different angles horizontally around a cylindrical school. The wave pattern is clearly seen except from perpendicular angles to the wave distance (plots 90 and 270). The closer the angle to perpendicular, the less linear the visible wave speed is.

Figure S19. A flash wave as recorded from different angles surrounding a cylindrical school around its length axis. The wave pattern is visible from every angle. In particular, when looking from below (angles of 240, 270 and 300 degrees), an anti-flash wave can be seen.

Figure S11. A flash wave as recorded from different angles horizontally around a ball-shaped school. The wave pattern is clearly seen and gets distorted the more we apply perpendicular angles to the wave distance (plots 90 and 270). Even in the perpendicular angles, we can see a dynamic of monotone growth/decay of the flashes.

Figure S12. A flash wave as recorded from different angles surrounding a ball shaped school around its length axis. The wave pattern is visible from every angle. In particular, when looking from below (angles of 240, 270 and 300 degrees), an anti-flash wave can be seen.

12 Flash waves in shoaling groups

In order to imitate a shoaling (disordered) group, we took a school consisting of 5k fish and placed it in different random directions (S22 and S23 Figs). It turned out that the fish density and the flash signal towards the observer are less significant than for an aligned school, but can still be clearly

seen. There is no clear flash change signal. The information-transfer signal (plot c. shows a clear wave of information). This can indicate that for a group of fish which is in a low level of alignment, the flash signal, as we defined it, is less useful for alarming about an upcoming event.

Figure S13. The four measurements for a shoaling scenario. The fish density and the observed flash signals are clear but less significant than in the aligned schools. The flash-change signal cannot be seen.

Figure S14. Another example of shoaling. Compared to S22 Fig, the patterns of the noises slightly differ but the signals are the same.

13 Flashes not due to evasive response

Not all the light reflection are caused by an evasive response of the fish. In this section, ‘noise’ is defined as the amount of visible flashes that are not caused by a reaction of the fish to a non-routine event, i.e. when the fish is obeying the regular Boids’ rules and the school is keeping its direction. Such noise can come from two sources: (i) Stable reflections when the ‘standard location of the fish is on the reflection plane between the observer and the light source, and (ii) Random movement of the fish that are not caused by the Boids’ rules.

Noise of type (i) can be caused when the fish is found ‘under’ a fish within the borders of the light distribution. I.e. if the fish stands ‘straight’ with its back fin pointed upwards and the light distribution is θ, an observer that is found within the θ range under the fish will see a reflection. In particular, the observer that is placed in the center of a school sees a cone of reflecting fish on top of it. If the observer is keeping the same depth but is staying away of the school, it does not see any flashes. Intermediate distances from the school leads to intermediate results. Fig 24a. shows the number of stable reflections the observer sees when it moves horizontally and “travels through” a center of a school of 1000 fish. When the observer is away of the school it can see (almost) no reflections. When it gets close to the school, the top of the school starts reflecting light and in the middle of the school the fish in the cone at the size of the reflection angle on top of the fish are reflecting light. In this case, the maximal number of reflecting fish is ~100 which is slightly less than expected if the school would be round. This appears as logical since the school’s shape is slightly oval.

Type (ii) noises derive from the noise in the motion of the fish. S24b,c Fig. show that the school's detectability depends on the noise in the roll axis and the position of the observer relatively to the school.

Figure S15. Noise sensitivity. a. The number of Boids reflecting light towards an observer which moves horizontally crossing the center of the mass of an oval group of 1000 "relaxed" Boids under light distribution resembles an ideal Snel's window. b. is a table of the average number of visible flashes towards a horizontal observer perpendicular to the school's swimming direction. The columns represent different noises in the roll of the fish and the rows show different noises in the direction of the velocity of the fish. The roll noises have stronger effects on the flashes. Flashes in the high velocity noise are also due to strong torsion (changes in roll) required to "correct" the noise. A similar analysis with roll always set to zero gave zero flashes in all configurations. c. shows the same scenario of plot b, except that the observer is placed facing the school's direction. The trend of dependency on the roll-noise is preserved. The combined effect of both noises has a stronger relative influence on the number of flashes and the total numbers of flashes are lower in comparison to each one of them separately.
