# Supplementary material for: Schooling of light reflecting fish
Source: PLoS One. 2023 Jul 21;18(7):e0289026. doi: 10.1371/journal.pone.0289026 (PMC10361475; doi:10.1371/journal.pone.0289026)
Supplement: Attachment — Submitted filename: Response to Reviewers.docx [file pone.0289026.s031.docx]

Dear Editor and Reviewers,

We thank the referees for their positive reviews and constructive comments. All comments have been addressed. We have accepted all the (minor) comments of Reviewer #1 and made changes accordingly. In particular, we redrew figure #3 in order to make it clearer. Regarding the resolution of the figures (cf. Reviewer #2), the resolution of the figures in the auto-generated PDF appeared to be lower compared to the resolution of the figures we originally submitted. Figures with a higher resolution will be generated per request from the production editor.

Once again, we sincerely appreciate the efforts of the reviewers in evaluating our manuscript. Their feedback has been instrumental in refining our work, and we hope that the revised version now meets the standards of PLoS One.

Yours sincerely,
Assaf Pertzelan, Moshe Kiflawi, and Gil Ariel
